# Wasting in under five children is significantly varied between rice producing and non-producing households of Libokemkem district, Amhara region, Ethiopia

**DOI:** 10.1186/s12887-019-1677-2

**Published:** 2019-08-28

**Authors:** Achenef Motbainor, Abeba Taye

**Affiliations:** 1 School of Public Health, College of Medicine and Health Sciences, Bahir Dar University, P. O. Box: 79, 1000 Bahir Dar, Ethiopia; 2 GAMBY Medical and Business College, Bahir Dar, Ethiopia

**Keywords:** Wasting, Malnutrition, Undernutrition, Under five children, Ethiopia

## Abstract

**Background:**

Acute undernutrition (wasting) is a condition in which a child becomes too thin for his or her height because of weight loss or failure to gain weight. Wasted children have greater risk of morbidity and mortality compared to their normal counterparts. There are significant number of children in Africa and Asia who suffered from all forms of malnutrition. This study aimed to determine the prevalence of wasting and its associated factors among 6–59 months of age children in Libokemkem district, Amhara region of Ethiopia.

**Methods:**

A community based cross-sectional study design was employed from June 1st to August 30th, 2017. A total of 876 households were selected using stratified multistage sampling technique. Interviewer administered structured questionnaire was used to collect socio demographic and other characteristics of the participants. Anthropometric data from the children was collected using the procedure stipulated by World Health Organization/United Nations International Children’s Emergency Fund. Kebeles, the smallest administrative unit of the country, were stratified in to two groups based on the presence and absence of rice production program. Then, the children were selected randomly from the households that have been included by using systematic random sampling technique. To assure the quality of data, pretest was done on 5.00% of the total sample size. Data were coded and entered using Epi Info version 7 software and exported to Statistical Package for Social Sciences version 20 software for further analysis. Bivariate and multivariate logistic regression analysis were employed to determine the significant association between independent and dependent variables. Binary logistic regression was run to identify candidate variable for multivariate logistic regression. Those variables with a *p*-value < 0.25 were entered in to multivariate analyses to check the association between independent and dependent variables. Significant association set at a *p* value < 0.05.

**Results:**

The total prevalence of acute malnutrition (wasting) was 7.10% and from this 2.50% were severely wasted. It was significantly higher among children in non-rice producing community at 11.80% (95% Confidence Interval (CI): 7.90, 13.88) than rice producing one at 3.34% (95% CI: 1.60, 5.65). Children whose mothers had no power to decide how income earned is used (Adjusted Odds Ratio (AOR) = 3.94, 95% CI: 2.12, 7.31), children who lived in areas with no rice production program (AOR = 3.16, 95% CI: 1.58, 6.33), children whose mother had no formal education (AOR = 3.64, 95% CI: 1.70, 7.79) were also significantly associated with wasting. Monthly income less than1500 Ethiopian birr (AOR = 4.14, 95% CI: 2.14, 7.99), presence of diarrheal disease for the last 15 days (AOR = 2.49, 95% CI: 1.34, 4.64) and complementary food starting before 6 months (AOR = 2.62, 95% CI: 1.26, 5.42) significantly associated with wasting.

**Conclusion:**

There was substantial difference between rice producing program and non-producing program communities with regarding to wasting. Children from rice producing program communities have better nutritional status than their counterparts. Intervention needs to be conducted on mother’s decision-making power over household income, mother’s education, and on the productive agricultural practices like improved rice producing programs.

## Background

Under-nutrition has persistently remained one of the greatest public health threats in the world for developing countries [1]. Wasting or acute malnutrition is one forms of undernutrition that is threatening life and resulted from hunger and/or disease. According to World Health Organization/United Nations International Children’s Emergency Fund (WHO/UNICEF)/World Bank estimates, in 2016, nearly 52 million or 7.70% of global under 5 children were wasted and from this 17 million were severely wasted [2]. At global level, more than 50% of childhood mortality in children under 5 years old triggered by acute malnutrition, which implies that about 3.5 million children die of malnutrition each year [3]. The majority of this problem is found in Africa 7.70%, Asia 9.90%, Oceania 9.40% [2]. Community based case-control study done among Nepal under five children showed that from the total participants 4.14% is severely wasted [4]. Another similar study done in Pakistan also showed that the prevalence of wasting was 16.20% [5].

The prevalence of wasting in Ethiopia despite recent economic progress, is among the worst in the world and it remains major public health problem. According to the 2016 Ethiopian Demographic and Health Survey (EDHS) report, 10% of children were wasted (30% severely wasted) at country level. Somalia and Afar region registered the highest prevalence of 23 and 18%, respectively [6]. In east and west Gojjam zones of Amhara region of Ethiopia, it was found to be 17.30% [7]. In North Shewa zone of Oromya region, Ethiopia, prevalence of wasting among preschool children was 16.70% [8]. In Bule Hora district of Oromya region and Hawassa zuria district, South Nation and Nationalities Regional Peoples, wasting was 13.40 and 23.60%, respectively [9, 10]. Other similar study conducted in Haramaya district, Oromya region of Ethiopia also showed that the prevalence of wasting was 10.70% [11]. The worst figure that showed the prevalence of wasting (28.20%) is found in Hawassa, South Nation and Nationalities of Ethiopia [12].

There are a number of factors associated with wasting. Socio-economic background, maternal education and health conditions, food availability, access to health services, infectious diseases, low birth weight, inadequate exclusive breast feeding, inappropriate complementary feeding practices, low nutritional knowledge and awareness, insufficient energy and micronutrient intake and birth spacing are some of the factors associated with child undernutrition, especially with wasting.

A study done in Bangladesh that used linear discriminant analysis to identify determinants of undernutrition showed that socio-economic and maternal health conditions were the two most factors associated with wasting [13]. Similarly, in Nepal, Pakistan and Iran studies showed that low socio-economic status of the household was the most significant determinant of acute malnutrition [4, 5, 14]. In Ethiopia, the same conditions associated with low socio-economic and wealth status were observed to be associated with wasting [9, 12, 15].

In different parts of the world, besides socio-economic conditions, other factors were also found to have significant effect on wasting including, birth interval, paternal education, breast feeding initiation time and family size [4, 16]. A community based cross-sectional study done in east and west Gojjam zones of Amhara region, and Haramaya district of Oromia showed that there is a significant association between wasting and food security status of the households [7, 17].

There are different short term and/or long-term health outcomes that resulted from malnutrition both in children and elderly. These include increased risk of morbidity and early mortality, delay physical growth and motor development, lower intellectual quotient, less social skill and greater behavioral problem, also high susceptibility to chronic diseases [18]. Generally, malnutrition increased the risk for death within each of the common comorbid conditions including ischemic heart disease, chronic obstructive pulmonary disorder, stroke or transient ischemic attack, heart failure, chronic kidney disease, and acute myocardial infarction [19].

Ethiopia has made a remarkable progress and achievements in the past decade in economic growth and health services. The country implemented several different interventions to improve households’ socio-economic status and nutritional status of preschool children. Libokemkem district is one of the areas in which these programs are implemented. It also has relatively high rates cash crops (maize, barely and rice) and modest livestock (sheep and cattle). But when rain fall is unusually abundant as recent years, and the district is affected by runoffs and flooding that make it difficult to rely on cash crops. Instead, income becomes mostly depending on selling livestock, which prevent them to using sheep and cattle for their food sources. It is assumed that whenever there is an improvement in the production capacity of the residents, they can participate in the market. This is also increase their income and the probability of using sheep and cattle for food will increase. In addition, residents have been given training about the importance of rice for food security of the household and how to use it as their source of food by mixing with other food items like meat, vegetable and others. It is believed that these things may change the nutritional status, feeding style and awareness of the community.

Therefore, this study aimed at determining the level of wasting and associated factors in children 6–59 months of age in kebeles which have the program for improved rice production and in those that do not.

## Methods

### Study setting

A community based cross-sectional study design was conducted from June 1st to August 30th, 2017 to determine the prevalence of wasting and its associated factors among children 6–59 months of aged in Libokemkem district, South Gondar zone of Ethiopia. The district is located at a distance of 645 km form Addis Ababa, the capital city of Ethiopia, and 80 km from Bahir Dar the main city of the Amhara region in northern Ethiopia. According to the Regional Bureau of Finance and Economy projection (2014), the study area had a total population of 220,688 (49% are female and 51% are male). Children under 5 years of age accounted 35,950. All 6–59 months old children paired with their mothers living in the randomly selected kebeles (the smallest administrative unit of the country) in the district and included in the study were taken as the study population. Randomly selected households in the selected kebeles were the sampling units and the required data were drawn from the included children their mothers or care givers.

### Sample size determination

The sample size was determined by using two approaches due to the fact that the study has two objectives. The first approach was by using single population proportion formula and the following assumptions; confidence level of 95%, margin of error or level of precision 5.00% and the prevalence of wasting 20.80% using the previous study [20]. Considering the above assumptions and 2 design effect, the estimated sample size was 276 children. For the second objective the sample was determined using 10% difference between the two kebeles (kebeles with rice production program and kebeles without the program). Assuming that the kebeles with no rice production program and no child feeding sensitization, will have 20.80% wasting and kebeles with the program will have 10.80% wasting, 80% power of study, 95% confidence level and design effect of 2 and Epi Info 7 software application, the calculated sample size was 876. This later sample size was greater than the first one and considered as sufficient to address both specific objectives and taken as final sample.

### Sampling technique or procedure

Stratified multistage sampling technique followed by systematic sampling technique was used to select kebeles. Stratification of the kebeles was done based on the presence and absence of rice production programs in the district. After the stratification, 6 kebeles (3 from each stratum) that satisfy 20–30% of the total district kebeles were selected systematically and included in the study. The households from each kebele were selected randomly based on sampling interval which was determined by dividing the total households of the kebele to proportionally allocated sample size of each kebele. The sample size for each kebele was determined after the total sample was divided proportional to households of the kebele. When there was more than one mother with under five children in the same household then one mother was selected by lottery method and when there was no eligible mother with under five children in the selected household, the next household was visited and replaced.

### Inclusion and exclusion criteria

#### Inclusion criteria

All children 6–59 months of age and whose parents lived in the selected kebeles at least 6 months prior to the data collection period.

#### Exclusion criteria

Children with serious illness or physical deformation which make anthropometric measurements difficult as were children who suffered with diarrhea and become dehydrated and children who had other diseases that might decreased the weight of the child.

#### Dependent variables

Wasting (Acute malnutrition)

### Operational definition

Wasting: Nutritional deficient state of recent onset related to sudden food deprivation or mal absorption utilization of nutrients which results weight loss, weight-for-height below − 2 Standard Deviation (SD) from the WHO median value [21].

Severe wasting: weight-for-height below or less than − 3 z-score for children under 5 years of age [21].

Acute respiratory illness: A child with cough and fast breathing or difficulty in breathing.

Complementary foods: Foods which are required by the child, at 6 months of age, in addition to breastfeeding so long as breast feeding is not sufficient.

Diarrhea: Loose stools for three or more times in a day and a sign of dehydration.

Family size: Total number of people living in the same house during the study period.

### Data collection tools and procedures

Interviewer administered structured questionnaire was prepared by reviewing available literature and other standard questionnaires that were already validated and used by EDHS, 2016 was used to collect data. The questionnaire had socio-demographic, socio-economic, environmental health facilities and child feeding practices of the community sections. Anthropometric data was collected by trained nurse data collectors using a measuring board with a head board and sliding foot piece and stadiometer to measure height/length and for weight salter scale using basin and standardized scales. It was collected using the procedure stipulated by the World Health Organization/United Nations Children’s Fund for taking anthropometric measurements [22]. There were two data collectors as a team and both of them taken the measurement during data collection time and the average was recorded using the questionnaire as raw data.

#### Weight

Weight of the child was measured by electronic digital weight scale with minimum/lightly/clothing and no shoes. Calibration was done before weighing each child by setting it to zero and by weighing a pre-known weight material.

#### Height

The height of the child was measured by two trained nurses. For those less than 2 years of age measurement was done without shoes and the height read to the nearest 0.1 cm by using a horizontal wooden length measuring board with the infant in recumbent position on a hard and flat surface. However, height of children 24 months and above was measured using a vertical wooden height board by placing the child on the measuring board, and child standing upright in the middle of board. The child’s head, shoulders, buttocks, and heels touching the board. Height (length) of the child was recorded to the nearest 0.1 cm.

### Data management and analysis

#### Data entry

The principal investigator and the supervisor monitored the overall data collection process by checking completeness and consistency of the required type of data and corrected faults on the spot. The investigator coded the questionnaire and entered the data in to Epi Info statistical software package. After the data entry data cleaning, was performed by running frequencies of each variable to check for accuracy and consistency.

### Data quality assurance

To ensure the quality of data, five data collectors and two supervisors were recruited and trained for 4 days on issues conducting interview, questionnaire content, the ethical aspect while approaching the care givers which was in a polite and respectful manner and on how to do anthropometric measurement. Data collectors were selected based on profession and previous experience of data collection. The questionnaire was pretested using 5.00% of the total sample in other kebele which were not included in the study. The English version questionnaire was translated to Amharic language and again translated back to English by experts who are fluent in both languages to check the consistency. After the data collection, the information was reviewed and errors were returned to the data collectors for correction on daily bases.

### Data analysis

The data were checked for its completeness and consistency then coded and entered in to a computer using Epi Info 7 and cleaned [23]. Anthropometric index weight-for-height z-score (WHZ) was analyzed by using WHO Anthro and categorized as wasted if WHZ < − 2 z-score and as normal if WHZ ≥ − 2 z-score. Extreme outlier like < − 5 z-score of WH was omitted from the analysis [24]. Finally, data were exported to SPSS version 20 for further analysis [25]. Descriptive analysis was used to compute descriptive data that used to describe the percentage and number of distribution of respondents by socio-demographic characteristics and other variables in the study.

Bivariate analysis for each factor was conducted to determine the candidate for further or multivariate analysis. All variables with a *p*-value < 0.25 in the bivariate analysis was entered to the next step [26]. Then multivariate analysis was conducted to assess the significant association between independent and dependent variables by controlling potential confounders. At this step, model fitness and the presence of multicollinearity were assessed. The model fitness was checked by observing the difference of -2log likelihood between the model with only the constant and with the predictors. Finally, 95% CI and adjusted odd ratio were used to report the significant variables associated with wasting. *P*-value less than 0.05 considered as statistically significant association.

### Ethical consideration

Ethical clearance was obtained from GAMBY Medical and Business College ethical review committee and Amhara Region Institute of Public Health. Official letter of cooperation was written to Amhara Regional State Health Bureau, Libokemkem district administrations, and health office. Further letter of cooperation from the district administration and health office were obtained and submitted to health centers, health posts and kebele chairman for facilitation and ethical issues. The nature of the study was fully explained to the study participants to obtain their oral informed consent prior to participation in the study. Informed consent was obtained from each respondent before the interview. Privacy and confidentiality of collected information was well kept at all level.

## Results

### Socio-demographic characteristics

A total of 862 women with young children 6–59 months of age were interviewed and gave complete responses with a response rate of 98.00%. Four hundred sixty-four households had a family size of six and more. Six hundred nine households lived in the rural area and the rest lived in urban. Forty three percent of the households of the study participant were in improved rice production kebeles of the study area. About 88.40% of Households (HHs) headed by males. The majority of the preschool children had parents who lived together (90%) and 7.2% had divorced.

The majorities of respondents were of the Amhara ethnicity group (98.80%) and (98.1%) were Ethiopian Orthodox Christians. Four hundred ninety-seven of the mothers and 467 of the fathers had no formal education. About 67 % (67.1%) of mothers and 70% of fathers were farmer. Six hundred eighty-nine mothers had the power to decide how the money they earn would be used. Almost 80 % (79.6%) of households owned farm land and 80.2% owned livestock (Table 1).

**Table 1:** Socio demographic characteristics of study participants Libokemkem district, Amhara region, Ethiopia, 2017

Characteristics/variables	Categories	Frequency (n)	Percent (%)
Family size	3	66	7.70
	4–5	332	38.50
	6 and above	464	53.80
Monthly income	Below 1500	334	38.70
	Above 1500	528	61.30
Ethnicity	Amhara	852	98.80
	Tigrie	4	0.50
	Oromo	6	0.70
Religion	Orthodox	846	98.10
	Protestant	4	0.50
	Muslim	12	1.40
Head of Household	Female	100	11.60
	Male	762	88.40
Ownership of farm land	No	176	20.40
	Yes	686	79.60
Ownership of Livestock	No	171	19.80
	Yes	691	80.20
Marital Status	Married	776	90.00
	Divorce	62	7.20
	Widowed	24	2.80
Place of residence	Rural	609	70.60
	Urban	253	29.40
Cluster	No Rice production program	491	57.00
	Rice production program	371	43.00
Maternal education	No formal Education	497	57.70
	Formal education	365	42.30
Paternal educational	No formal Education	467	54.20
	Formal education	395	45.80
Maternal occupational	Farmer	578	67.10
	Merchant	284	32.90
Paternal occupational	Farmer	603	70.00
	Merchant	162	18.80
	Government employ	97	11.30
Maternal Decision Power	No	173	20.10
	Yes	689	79.90

### Child characteristics

From the total 862 children included in the study, 449 were males and the rest were females. Five hundred sixty-two children were in the age group of 6–23 months. Forty two point 3 % of children had diarrhea in the last 15 days prior to data collection time (Table 2).

**Table 2:** Child characteristics of study participants among 6–59 month of age children in Libokemkem district, Amhara region, Ethiopia, 2017

Characteristics	Categories	Frequency (n)	Percent (%)
Child sex	Female	413	47.90
	Male	449	52.10
Child age	6–23	562	65.20
	24–59	300	34.80
Fever	No	735	85.30
	Yes	127	14.70
Diarrhea	No	497	57.70
	Yes	365	42.30
ARI	No	488	56.60
	Yes	374	43.40
Measles	No	694	80.50
	Yes	168	19.50
Oedema	No	862	100.00

### Maternal characteristics and caring practice

Five hundred seventy-one mothers were in the age group of 30 and below years. Seven hundred fifty-five mothers visited health facilities for Antenatal Care (ANC) services during their pregnancy. Four hundred fifty-six mothers give birth at a health center and 729 mothers were assisted by health professionals during delivery. Fifty six percent of mothers had awareness about family planning and 53.10% used it. Four hundred one mothers breast feed their children immediately after birth and 217 had a pre-lacteal food or fluid practices. Almost 90 % (90.30%) of mothers exclusively breast fed the child and 88.30% of mothers gave additional food at six-month (Table 3).

**Table 3:** Maternal characteristics and caring practice of study participants in Libokemkem district, Amhara Region, Ethiopia, 2017

Characteristics/variables	Categories	Frequency (n)	Percent (%)
Maternal Age in years	≤ 30	571	66.20
	> 30	291	33.80
Total number of children	1	149	17.30
	2–3	362	42.00
	4 and more	351	40.70
Total no of under five children	1	449	52.10
	2–3	413	47.90
ANC visit	No	107	12.40
	Yes	755	87.60
Exclusive breast feeding	Before 6 months	84	9.70
	6 months and above	778	90.30
Initiate of breast feeding	Before 1 h	401	46.50
	1–24 h	357	41.40
	After 1 day	104	12.10
Pre- lacteal food or fluid	No	645	74.80
	Yes	217	25.20
Additional food	Below 6 months	101	11.70
	At 6 months and above	761	88.30
Gestational age at birth	Before 9 months	143	16.60
	At 9 months	719	83.40
Child Immunization Status	No	16	1.90
	Yes	846	98.10
Vaccine Received	BCG only	14	1.60
	Penta valent	137	15.90
	Measles	183	21.20
	All vaccines	528	61.30
About Family planning	No	379	44.00
	Yes	483	56.00
Family planning used	No	404	46.90
	Yes	458	53.10
Birth order	1	155	18.00
	2–3	372	43.20
	4 and above	335	38.90
Place of delivery	At home	133	15.40
	Health post	63	7.30
	Health center	456	52.90
	Hospital	210	24.40

### Associated factors and their difference between the two areas

Table 4 depicts that significance difference between the two areas are seen only for household monthly income and handwashing frequency variables otherwise no differences for all other variables.

**Table 4:** Associated factors and their difference status between the two areas using chi-square test, Libokemkem district 2017

Variables	Frequency (%)			*P*-value
	Program area	Nonprogram area	Total	
Maternal education				
No formal education	208 (41.85)	289 (58.15)	497	0.41
Formal education	163 (44.65)	202 (55.35)	365	
Decision making power				
No	76 (43.93)	97 (56.07)	173	0.79
Yes	295 (42.82)	394 (57.58)	689	
Monthly income				
< 1500 Ethiopian birr	130 (38.92)	204 (61.08)	334	0.05
≥ 1500 Ethiopian birr	241 (45.64)	287 (54.36)	528	
Child sex				
Female	185 (44.79)	228 (55.21)	413	0.32
Male	186 (41.43)	26,358.57)	449	
Child age				
6–23 months	232 (41.28)	330 (58.72)	562	0.15
24–59 months	139 (46.33)	161 (53.67)	300	
Complementary feeding initiation				
< 6 months	42 (42.58)	59 (58.42)	101	0.75
≥ 6 months	329 (43.23)	432 (56.77)	761	
Presence of diarrhoea				
No	220 (44.27)	277 (55.73)	497	0.40
Yes	151 (41.37)	214 (58.63)	214	
Hand washing frequency				
< 4	105 (33.76)	206 (66.24)	206	0.00
≥ 4	266 (48.28)	285 (51.72)	285	

### Magnitude of acute malnutrition

The mean WHZ-score of 6–59 months of aged children based on WHO Anthro software was found to be 1.75. According to the WHO reference standard taking with − 2 SD as cutoff point, 7.10% of study children fell below − 2 SD and out of this, 2.50% was severely wasted (WHZ-score < − 3). The prevalence of wasting in areas with non-rice producing program was 11.80% (95% CI: 7.75, 13.68) and areas with rice producing program was 3.34% (95% CI: 1.67, 5.65). The prevalence of wasting and severely wasting was higher in male children (7.3%) and 2.6%, respectively, than female that was 7.00% wasted and 2.40% severely.

#### Factors associated with wasting among 6–59 months of age children

In bivariate binary logistic regression analysis, it was found that the following socio demographic and economic factors were significant: maternal educational status, decision making power of the mother, owner ship of land and monthly income. Child age, child sex and the presence of diarrhea for the last 15 days prior to the survey were also selected for further analysis based on the criteria. In addition to this from maternal characteristics and caring practices age of complementary food initiation and from environmental health condition frequency of hand washing practices were identified variables as the candidate for multivariate analysis at *p*-value less than 0.25 and those variables whose *p*- value less than 0.05 were considered as significantly associated with wasting.

In the multivariate logistic regression analysis, cluster, child sex, child age, decision making power of the mother, presence of diarrhea for the last 15 days prior to the survey, maternal education, monthly income and age at complementary food initiation were identified as factors significantly associated with wasting. The odds of children with in age group of 6–23 months was 3.34 times higher (AOR = 3.34, 95% CI: 1.47–7.59) to be wasted than the odds of children in 24–59 months of age group. The odds of children whose mothers had no formal education was 3.58 times higher (AOR = 3.58, 95% CI: 1.75, 7.32) to be wasted than the odds of children whose mothers had formal education.

The odds of children from households with low monthly income was 3.30 times higher (AOR = 3.30, 95% CL: 1.54, 7.12) to be wasted than the odds of children from households with high monthly income. Similarly, the odds of children in the non-rice production cluster or area was 3.16 times higher (AOR = 3.16, 95% CI: 1.58, 6.33) to be wasted than the odds of children who lived in the rice production cluster. It was also found that, the odds of children whose mother had no power to decide how the money earned will be used in the household was 3.89 times higher (AOR =3.89, 95% CI: 2.14, 7.10) to be wasted than the odds of children whose mother had power to decide how the money earned will be used.

The odds of being wasted in male children were 2.44 times higher (AOR = 2.44, 95% CI: 1.3, 4.57) than female children. The odds of children who had diarrhea in the past 2 weeks were 2.25 times higher (AOR = 2.25, 95% CI: 1.25, 4.05) to be wasted than children who had no diarrhea in the past 2 weeks. Child age at complementary food initiation also found to be another significantly associated variable with wasting. The odds of children who were start their complementary food before 6 months was 2.32 times higher (AOR = 2.32, 95% CI: 1.15, 4.70) to be wasted than children who were start their complementary food at 6 months and above (Table 5).

**Table 5:** Bivariate and multivariate logistic regression analysis of factors associated with wasting among 6–59 months of age children, Libokemkem district, 2017

Explanatory variables	Wasting		Crude Odds Ratio COR (95% CI)	AOR (95% CI)
	Yes	No		
Clustered				
Area with no rice production program	51	439	3.48 (1.83–6.62)	3.16 (1.58–6.33) **
Area with rice production program	12	359	1	1
Maternal Education:				
No formal education	52	444	3.77 (1.94–7.33)	3.58 (1.75–7.32) **
Have formal education	11	354	1	
Decision making power:				
No	30	143	4.16 (2.46–7.05)	3.90 (2.14–7.10) **
Yes	33	655	1	
Monthly income:				
Below 1500	47	286	5.26 (2.93–9.44)	3.31 (1.54–7.14) **
Above 1500	16	512	1	
Child sex:				
Female	17	396	1	
Male	46	402	2.67 (1.50–4.73)	2.45 (1.31–4.57) **
Child age:				
6–23	55	8	3.97 (1.86–8.45)	3.34 (1.47–7.59) **
24–59	8	292	1	
Presence of Diarrhoea				
No	23	473	1	
Yes	40	325	2.53 (1.49–4.31)	2.25 (1.25–4.05) **
Age at complementary food start				
Below 6 months	16	85	2.86 (1.55–5.26)	2.32 (1.15–4.70) **
At 6 months and above	47	713	1	
Hand washing frequency				
3 times and below per day	40	270	3.40 (2.00–5.80)	1.25 (0.60–2.58)
4 times and above per day	23	528	1	

** = *P* < 0.01

## Discussion

Interventions implemented to address some problems in the community may have additional outcomes than the primary objectives of the program. In this regard agricultural interventions that have been implemented to enhance the productivity of the community can make a positive contribution to public health nutrition improvements. This is because the packages included in the agricultural productivity are highly linked with child nutrition. Literature demonstrates that there are associations between agricultural interventions and nutritional outcomes [27]. In the same way, this differences in child nutritional status between the two areas might be resulted from this agricultural intervention. The production of targeted nutrition rich crops, homestead gardens and diversification of agricultural production systems towards fruits and vegetables and aquacultures can potentially improve nutrient intake and nutritional outcomes [27]. Although the current intervention is focusing on rice production and does not include improvements in other types of agricultural products, it shows that when the economic status of the community is improved, the improvements extend to child nutrition.

Food security does not always guarantee nutritional security by its own, but it can be a precursor for nutritional improvements so long as the household properly managed the available foods. Other research has shown that there tend to be nutritional improvements when the households become foo secured and have better economic status [28].

Besides the effects of the rice production program, this research has also identified other factors associated with wasting. As supported by other studies, maternal education was found to be significantly associated with wasting [29]. It is expected that when level of maternal education is improved, all types of child care practices could improve including child feeding practices. Moreover, educated mothers can change traditional beliefs like disease causation, improve breastfeeding, attitudes and practices and more easily apply the information they get from different intervention programs.

Maternal decision-making power over the income of the household was the other factor associated with wasting. This variable is linked with different aspects of the household that have direct or indirect relationships with nutritional status of children including household food security, women’s empowerment and socio-economic status [30]. The finding is supported by another study done in Ghana that evaluate the contribution of women’s empowerment in agricultural productivity and child nutrition, which also found that women’s empowerment is strongly associated with the quality of infant and young child feeding practices [31]. Therefore, nutrition improvements associated with this intervention might resulted from both child care practices and household economic improvements which in turn result from women’s empowerment and control over decision-making in the household. Improvements in infant and child feeding practice as means of nutrition improvement was also observed in this research. Those children who start their complementary food at 6 months were less likely to be wasted than children who started their complementary food before 6 months.

Children who were suffering from diarrhea within the past 2 weeks of the survey day were more likely to be wasted compared with children who had no diarrhea diseases. It is a well-established fact that children suffering from diarrhea are more at risk to be wasted than their counter parts [15]. Here diarrhea is mentioned as associated factors with wasting in order to give emphasis when designing intervention like that of agricultural productivity improvements. When designing any sort of program, integrated intervention needs to be included to address this health problems which has a direct effect on the nutrition status of children.

## Limitation of the study

There might be potential recall bias among respondents when they are answering questions related to past events. Moreover, being cross-sectional, the study did not address seasonal variations of child nutritional status.

## Conclusion

Wasting was highly prevalent problem in the none rice production area. Having rice production as a program as well as maternal education, decision making power of the mother on the household income, monthly income, presence of diarrhea and complementary food initiation time were factors significantly associated with wasting. Intervention should focus on expanding the program for better production of rice cultivation in other kebeles to improve the income by strengthening women empowerment and saving at HHs like credit and saving process with collaborate of stake holders. Nutrition improvement proved to be an important outcome of strengthening agricultural productivity and programs targeting agricultural productivity would be well served by targeting nutrition improvement even more directly.

## Data Availability

All data generated or analyzed during this study are included in this published article. Anyone who wants the data set for educational or other purpose can obtain from the corresponding author by e-mail.

## Abbreviations

ANC: Antenatal care
AOR: Adjusted odds ratio
ARI: Acute respiratory tract infection
BF: Breast feeding
CI: Confidence interval
COR: Crude odds ratio
EDHS: Ethiopian demographic and health survey
SD: Standard deviation
SPSS: Statistical package for social sciences
SSA: Sub Saharan Africa
WHO/UNICEF: World Health Organization/United Nations International Children’s Emergency Fund
WHZ: Weight-for-height Z-score
